# Tumor Suppressor Gene-Based Nanotherapy: From Test Tube to the Clinic

**DOI:** 10.1155/2011/465845

**Published:** 2011-01-24

**Authors:** Manish Shanker, Jiankang Jin, Cynthia D. Branch, Shinya Miyamoto, Elizabeth A. Grimm, Jack A. Roth, Rajagopal Ramesh

**Affiliations:** ^1^Department of Thoracic and Cardiovascular Surgery, The University of Texas of MD Anderson Cancer Center, Houston, TX 77030, USA; ^2^Department of Pathology, The University of Oklahoma Health Sciences Center, Oklahoma City, OK 73104, USA; ^3^Department of Experimental Therapeutics, The University of Texas of MD Anderson Cancer Center, Houston, TX 77030, USA; ^4^Peggy and Charles Stephenson Oklahoma Cancer Center, The University of Oklahoma Health Sciences Center, Oklahoma City, OK 73104, USA; ^5^Graduate Program in Biological Sciences, The University of Oklahoma Health Sciences Center, Oklahoma City, OK 73104, USA

## Abstract

Cancer is a major health problem in the world. Advances made in cancer therapy have improved the survival of patients in certain types of cancer. However, the overall five-year survival has not significantly improved in the majority of cancer types. Major challenges encountered in having effective cancer therapy are development of drug resistance by the tumor cells, nonspecific cytotoxicity, and inability to affect metastatic tumors by the chemodrugs. Overcoming these challenges requires development and testing of novel therapies. One attractive cancer therapeutic approach is cancer gene therapy. Several laboratories including the authors' laboratory have been investigating nonviral formulations for delivering therapeutic genes as a mode for effective cancer therapy. In this paper the authors will summarize their experience in the development and testing of a cationic lipid-based nanocarrier formulation and the results from their preclinical studies leading to a Phase I clinical trial for nonsmall cell lung cancer. Their nanocarrier formulation containing therapeutic genes such as tumor suppressor genes when administered intravenously effectively controls metastatic tumor growth. Additional Phase I clinical trials based on the results of their nanocarrier formulation have been initiated or proposed for treatment of cancer of the breast, ovary, pancreas, and metastatic melanoma, and will be discussed.

## 1. Introduction

Cancer is a major health problem in the world. In 2009, about 1,479,350 people living in the United States of America (USA), have been diagnosed with cancer [1]. About half of these cancer patients will die of the disease. The lifetime risk of developing cancer is predicted to be 1 in 2 for men and 1 in 3 for women [1]. Dissemination of scientific information and cancer awareness have reduced the incidence for certain cancer types while the incidence for other cancer types remain unchanged or increased. For example, reduced incidence of lung cancer in men due to cessation of smoking has been observed while the lung cancer incidence in women is increasing. Similarly, ignoring the risks of exposure to ultraviolet rays and the potential for developing skin cancer has resulted in steady increase in the incidence of melanoma. 

 Effective cancer therapies developed in recent years have improved the survival of patients diagnosed with cancer. However, the overall five-year survival rate of cancer patients remain dismal and is less than 15% at least for solid tumors of epithelial origin [2]. Factors contributing to the poor survival rate despite having developed novel therapies include development of resistance to therapy by cancer cells, poor drug distribution and accumulation in the tumor, and nonspecific cytotoxicity to normal tissues thereby limiting the drug dosage. Thus, there is a tremendous effort in developing new cancer therapeutics that are efficacious and safe with minimal cytotoxicity to normal tissues. Testing and demonstration of such new therapeutics in preclinical studies will ultimately lead to testing in humans as a cancer drug.

One therapeutic approach that has shown promise and safety is cancer gene therapy [3]. The gene therapy approach that has exploded and tested widely in the last decade is the use of tumor suppressor genes (TSG's). Cell division and cell growth are tightly controlled processes often regulated by TSG's. However, alterations such as mutations, deletions, and silencing at the DNA, RNA, or protein level of TSG result in dysregulation of the cell growth and transformation [4]. Retinoblastoma (Rb) and p53 TSG are classical examples whose function when lost or altered has been shown to initiate or contribute to cell transformation [5, 6]. Furthermore, p53 gene mutations are observed in a majority of human cancers, suggesting it is an important gatekeeper of the cell. Apart from Rb and p53, several other TSGs have been identified and shown to regulate diverse cellular processes and loss of their function affects normal cell activity. Based on these observations, it was hypothesized that restoration of normal TSG function will inhibit cell proliferation and growth leading to cell death. Thus TSG-based cancer therapy was conceived and initiated.

Early studies using viral vectors demonstrated that delivering TSG's resulted in tumor inhibition in animal models [3] (see Table 1). Translating these findings to the clinic demonstrated clinical and/or biological response to therapy. Stabilization of the disease (SD) was frequently observed in patients receiving therapy, and in few cases complete response to therapy as evidenced by tumors' regression [7–10]. Despite the encouraging clinical results observed in virus-based cancer gene therapy studies, this treatment strategy has limited application due to the elicitation of host-immune response by viral proteins [11–14]. Additionally, testing of virus-based cancer gene therapy for treatment for metastatic disease has not been proven to be successful so far. 

To overcome the limitations encountered with virus-based cancer therapy, several laboratories including our own laboratory have been testing nonviral-gene-delivery vehicles for cancer gene therapy. The nonviral vectors are of different composition and formulations. They also vary in their size and geometry. A majority of these nonviral vectors are nanometer (nm) in size and often have a lipid component. According to the National Cancer Institute (NCI), any biological or synthetic material which in any one dimension is less than 1 micrometer (*μ*m) is called a nanoparticle. Based on this definition, several nonviral vectors that are less than 1 *μ*m in size are referred as nanoparticles, nanocarriers, nanosomes, and so forth. 

An advantage of using nanoparticles as gene-delivery vehicles is that they can deliver therapeutic genes to *in situ* tumors that are disseminated inside the body [3, 15]. Studies have demonstrated nanoparticle-based gene-delivery results in antitumor activity in experimental preclinical tumor models. An added advantage of using nonviral nanocarrier systems, apart from the ease of manufacturing, is the avoidance of problems frequently encountered with adenovirus [15, 16]. 

In this paper, we will discuss our experiences with a lipid-based nanocarrier that was initially tested in the laboratory as a tumor suppressor gene-delivery vehicle and later tested in the clinic for the treatment of nonsmall cell lung cancer (NSCLC). Plans for applying our nanocarrier-based cancer gene therapy technology for treatment of other solid cancers will also be discussed.

## 2. Gene-Based Nanotherapy

### 2.1. Laboratory Studies

Our interest in testing lipid-based nanocarriers as gene-delivery vehicles arises from the following observations: (1) cancer is often metastasized in patients at the time of their initial diagnosis [1, 2]; (2) conventional therapies are ineffective in treating metastatic disease [17, 18]; (3) our own laboratory studies demonstrate that virus-based (retrovirus and adenovirus) tumor suppressor gene therapy for systemic therapy of metastatic cancer was ineffective; (4) preclinical studies demonstrated that nonviral vectors can deliver genes and drugs to localized and disseminated tumors [19–21]. 

Although several lipid-based nanocarriers were reported in the literature to be efficient gene-delivery vehicles, most of these studies were restricted to *in vitro* testing with few being tested *in vivo* [22–27]. Furthermore, only a limited number of nanocarriers has moved beyond the laboratory and has been tested in the clinic (see Table 2). The reasons for their inability to test several nanocarriers in the clinic are multifactorial and include inability to produce clinical grade nanocarriers in large quantities, inflammatory response [28–30], poor stability and short half-life of the nanocarrier *in vivo *[31, 32], interaction with serum proteins and aggregation [33, 34], poor uptake of the nanocarrier by the tumors, and rapid clearance by macrophage and the reticuloendothelial system (RES) [35]. 

Methods to overcome some of these limitations included PEGylation of the nanocarriers using polyethylene glycol (PEG). Pegylated nanocarriers demonstrated improved stability *in vivo*, reduced RES clearance, and increased accumulation in tumors resulting in enhanced antitumor activity [36–38]. Similarly, studies using neutral or negatively charged nanocarriers have reported effective delivery of oligonucleotides, siRNA, and chemotherapeutic drugs [39, 40]. Despite the advances made with neutral and anionic lipid-based nanocarriers, they have not been developed and tested widely as tumor-suppressor gene-delivery vehicles for cancer therapy.

In 1998, Templeton et al. [41] reported that cationic DOTAP:cholesterol (DOTAP:Chol) lipid nanocarrier efficiently delivered plasmid DNA to the lung when administered intravenously. Findings by Gaensler et al. [42] concurred that DOTAP:Chol lipid nanocarrier to be an efficient gene-delivery vehicle. Crook et al. [43] reported that inclusion of cholesterol was important and a key to achieving stabilization of the DOTAP:Chol-nanocarrier and efficient gene transfer. The key feature that makes this nanocarrier better than previously tested lipid-based nanocarriers is its stability and reduced interaction with blood proteins *in vivo* which is contributed by the inclusion of cholesterol [41]. Another key feature that likely contributes to its effectiveness is that the lipid-nanocarrier, when mixed with DNA, forms unique bilamellar vase-like structures that keep the DNA intact from rapid degradation [41]. However, it is likely that additional factors that are unknown at the present time may contribute to its effectiveness. 

Based on these reports, we initiated preclinical studies in our laboratory and tested whether DOTAP:Chol-lipid nanocarrier could efficiently deliver tumor suppressor genes when administered systemically and control metastatic lung tumors. Size fractionation studies showed our lipid nanocarrier was 200–400 nm in size and had a positive charge of 40 ± 2 mV [44, 45]. The nanocarriers are stable +4°C for over a period of one month when stored as empty nanocarriers and for at least 48 h when mixed with DNA. Although one may argue that our nanocarriers are large, results from our studies, as discussed below, support particle size of 200–400 nm to be optimal and to strike a balance between tumor uptake and macrophage clearance. Furthermore, we believe that the size of the nanocarrier will need to be varied and optimized depending on the disease to be treated and that the concept of one-size-fits-all disease treatments cannot be applied. 

*In vitro* studies showed transfection efficiency mediated by the nanocarrier varied among cell types that correlated with transgene expression [44, 46, 47]. Transgene expression was observed to be detectable as early as 12 h after transfection and was detectable up to 72 h after transfection albeit expression levels decreased over time. The transfection efficiency and transgene expression were observed to be consistent in a given cell line even when different tumor suppressor genes or marker genes were used. One factor that affected transfection efficiency and transgene expression was the size of plasmid contained in the nanocarrier. In general, a nanocarrier containing a plasmid that was 3-4 Kb in size produced higher transfection compared to nanocarrier containing a plasmid that was greater than 4 Kb in size. Furthermore, the DNA-containing nanocarrier was stable for at least 48 h when stored at +4°C and produced comparable transfection efficiency and transgene expression in tumor cells when compared to that produced by cells treated with a freshly prepared DNA-containing nanocarrier.

*In vivo* studies were initially focused on biodistribution and toxicity of the DNA-containing nanocarrier in immunocompetent mice. Biodistribution studies showed that the DNA-nanocarrier primarily localized to the lung when injected intravenously. However, over time the nanocarrier exited the lung and was detectable in other organs (liver, spleen, kidney etc). Toxicity studies demonstrated a dose-dependent response with LD10 being in the range of 55–70 *μ*g of DNA in the lipid nanocarrier and depended on the backbone of the plasmid DNA. The therapeutic gene contributed very little to toxicity (unpublished data).

We next investigated the therapeutic effects of a TSG-containing nanocarrier on human lung tumor xenografts established in nude mice. Marker gene expression showed marked transgene expression when injected intratumorally into subcutaneous lung tumor xenografts [40]. Efficacy studies showed that a TSGs-containing (p53, Fhit, Fus1, TSG101, or IL-24) nanocarrier, when administered intratumorally, produced significant growth inhibition compared to tumor growth inhibition produced by nanocarrier treatment containing control plasmid DNA [44, 46, 47]. Growth inhibition, produced by TSG-containing nanocarrier was independent of the tumor model, as comparable growth-inhibitory effects were observed in human H1299 lung tumor and murine UV2237 tumor xenografts established in nude mice and C3H mice, respectively [46]. Furthermore, repeated treatments showed greater tumor-growth inhibition that correlated with increased transgene expression when compared to growth-inhibitory effects produced by single treatments. Our study also showed that the therapeutic effect produced by p53 TSG-containing nanocarrier treatment was independent of the endogenous p53 status of the treated tumor. Additionally, the therapeutic effect produced using various TSGs was comparable, albeit differences existed among tumor types. These results provide evidence and support intratumoral treatments of localized tumors such as cancer of the head and neck that are unresectable with TSG-containing nanocarrier. It is envisioned that such localized intratumoral treatments with TSG-containing nanocarrier will reduce the tumor burden and make the tumor accessible to surgery and radiation therapy.

Since our objective and goal was to test the nanocarrier as a systemic gene-delivery vehicle for treatment of metastatic disease, we conducted *in vivo* studies using experimental tumor-metastasis models. Human H1299 (p53 null) and A549 (p53 wild-type) tumor cells were injected intravenously via tail vein to establish experimental lung metastasis in SCID/Beige and nude mice, respectively. Mice received daily intravenous treatments with a p53 TSG-containing nanocarrier for a total of six doses. At four weeks after the last treatment mice were euthanized, lungs were harvested and examined for the number of pulmonary nodules. A significant reduction in the number of pulmonary tumor nodules were observed in mice receiving p53 TSG nanocarrier treatment compared to the number of pulmonary tumor nodules in mice receiving control DNA-containing nanocarrier treatment [44]. Histopathological examination of the lungs from mice receiving p53 TSG-containing nanocarrier treatment showed few tumors with evidence of tumor cells undergoing apoptotic cell death compared to the number of tumors in the lungs of control mice and very few tumor cells undergoing apoptosis. 

Since the six-day treatment with p53 TSG-containing nanocarrier did not completely abolish pulmonary tumor growth we next determined whether these tumors will regrow and if they can be treated with a second cycle of treatment akin to that practiced in the clinic. For this purpose, mice bearing experimental lung tumors were divided into two groups. One group of mice (*n* = 8) received the initial six treatments with p53 TSG nanocarrier (day 1–6). A second group of mice (*n* = 8) received the initial six treatments with p53 TSG nanocarrier (day 1–6) and a second cycle of six treatments starting on day 30 (day 30–35). Mice (*n* = 4) from each group were euthanized on day 28 and on day 42. Lungs were harvested from the euthanized mice and the number of lung tumor nodules counted. Our results showed that a greater reduction in the number of pulmonary tumor nodules in the lungs of mice receiving two cycles of p53 TSG nanocarrier treatment compared to the reduction in tumor nodules in the lungs of mice receiving single cycle of p53 TSG nanocarrier treatment (unpublished data). Our results demonstrate repeated cycles of treatment are feasible and that they produced a greater therapeutic effect. 

We next determined the therapeutic effects of p53 TSG-containing nanocarrier in disseminated tumor mouse model. Injection of H1299 lung tumor cells into SCID/Beige mice results in disseminated tumors in various organs [44]. Treating these mice with the p53 TSG nanocarrier intravenously resulted in prolonged animal survival compared to survival of control mice that were either untreated or treated with a control plasmid DNA-containing nanocarrier [44].

Effective gene-delivery mediated by the nanocarrier was not restricted to p53 TSG therapy or to lung tumor models. Delivery of Fhit and Fus1 TSGs, that are frequently lost in human lung cancer, produced therapeutic effects that were similar to the therapeutic effects observed with p53 [44, 47]. Furthermore, combination of Fus1-containing nanocarrier with chemotherapy was shown to produce additive to synergistic therapeutic effect [48]. Similarly, systemic therapy with IL-24-containing nanocarrier inhibited human lung tumor and murine fibrosarcoma growth established in nude mice and immunocompetent C3H mice, respectively [46]. In all of these studies repeated treatments resulted in additive increases in transgene expression in the tumors with minimal expression in normal tissues adjacent to the tumor [49], a finding that was in contrast to the report by Li et al. [50] who showed repeated treatments reduced transgene expression due to induction of treatment-related inflammatory response. The differences in the outcomes were due to difference in the animal models used. We demonstrated that mice bearing tumors produced immunosuppressive factors within the tumor microenvironment that altered the host immune pathology resulting in no inhibitory effects on transgene expression [51]. Additionally, nanocarrier tracking studies demonstrated tumors that were larger in size had more nanocarriers compared to tumors that were smaller in size [49]. The uptake of the nanocarriers involved tumor-mediated phagocytosis. Furthermore, the inflammatory response produced in the tumor-bearing mice was markedly reduced. On the contrary, if the mice did not bear any tumors then the nanocarrier was widely distributed in the lung, and induction of treatment-related inflammatory response and shutting down of transgene expression following repeated treatments was observed [44, 49, 52]. Thus, the outcomes of repeated nanocarrier treatment and transgene expression can be regulated by the host pathology and disease conditions and therefore need to be considered during drug development. 

**Figure 1:**
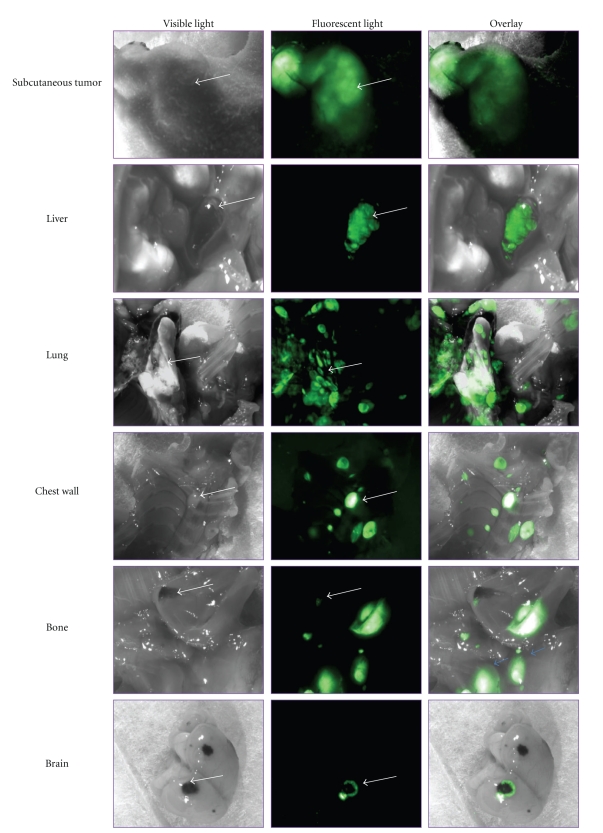
Detection of melanoma metastasis by fluorescent imaging. Human melanoma MeWo cell line was stably transfected and selected for GFP positive clones. The MeWo-GFP cells (5 × 10^6^) were injected intravenously via tail vein in nude mice. Mice were euthanized after four to five weeks and observed under normal white light and under fluorescent light. Melanoma tumors were detectable under white light in various organs. However, an increased number of GFP-positive tumors (green fluorescence) were observed under fluorescence light in various organs indicating melanoma metastasis. Additionally, tumors not visible under white light were detected by fluorescence.

**Figure 2:**
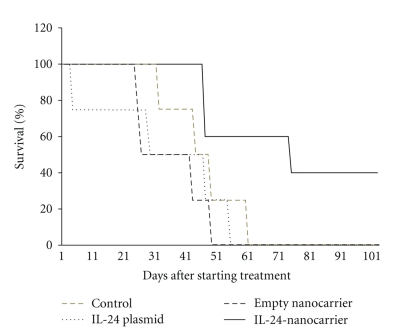
IL-24 nanotherapy improves animal survival. Nude mice were injected with MeWo-GFP. Ten days after tumor cell injection mice were divided into four groups: group received no treatment; group 2 received IL-24 plasmid DNA; group 3 received empty nanocarrier; group 4 received IL-24-containing nanocarrier (50 *μ*g DNA). Treatment was twice a week and administered intravenously for six weeks. Mice were monitored for animal survival. Mice receiving IL-24-containing nanocarrier therapy showed increased survival compared to all other treatment groups.

More recently, we have tested the systemic therapeutic effects of IL-24-containing nanocarrier treatments in a metastatic melanoma model. Nude mice injected with human melanoma (MeWo) tumor cells that are genetically modified to express the green fluorescent protein (GFP) produced tumors that metastasized to the lung, liver, brain, and several other organs and visible under bright light and fluorescent light (Figure 1). Mice injected with MeWo-GFP cells and bearing experimental metastasis were divided into the following groups: no treatment; treatment with IL-24 plasmid DNA; treatment with empty nanocarrier; treatment with IL-24-containing nanocarrier. Mice were treated twice a week (50 *μ*g DNA) until the study was terminated. Treatment of these mice having experimental metastasis intravenously with IL-24-nanocarriers resulted in prolonged animal survival compared to survival of mice that received other treatments or no treatment (Figure 2). These studies showed systemic treatment with our nanocarrier delivers therapeutic genes and produces effective anticancer activity.

**Figure 3:**
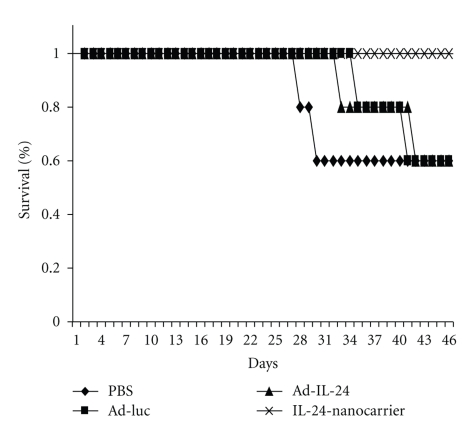
IL-24 nanotherapy for ovarian cancer. MDAH2774 (1 × 10^6^) cells were injected into the peritoneal cavity of nude mice. Mice were then divided into groups and treated with IL-24-containing nanocarrier intraperitoneally (i.p.). Mice that were treated with phosphate buffered saline (PBS), treated with adenovirus (Ad)-IL-24, or treated with Ad-luciferase (Luc) served as controls. An increase in animal survival was observed in mice that received IL-24-containing nanotherapy compared to all other treatment groups.

We next determined whether our nanocarrier can deliver TSGs to ovarian cancer when administered intraperitoneally (i.p.) and whether it was superior to adenovirus-mediated gene-delivery in producing a therapeutic effect. Nude mice were injected into the peritoneum with human ovarian MDAH2774 tumor cells. The mice rapidly form ascites with disease progression and at which time if untreated they will have to be euthanized. These i.p. tumor-bearing mice were divided into groups and treated as follows: treated with IL-24-containing nanocarrier, treated with adenovirus (Ad)-IL-24, treated with Ad-luciferase (Luc), or treated with phosphate buffered saline (PBS). Animals were monitored daily and animal survival recorded. As shown in Figure 3, mice receiving IL-24-containing nanocarrier showed a trend for increased survival compared to all other treatment groups. Our preliminary results showed nanocarrier-based therapy was more effective than adenovirus-based therapy in controlling tumor growth and progression for ovarian cancer. Finally, our studies demonstrate DOTAP:Chol-based nanocarrier is efficient in delivering therapeutics genes to local and metastatic tumor sites and can be administered via various routes resulting in enhanced therapeutic effects in preclinical models.

### 2.2. Clinical Studies

On the basis of our preclinical studies, a Phase I clinical trial for the systemic treatment of nonsmall cell lung cancer (NSCLC) has been initiated at the University of Texas MD Anderson Cancer Center, Houston, Texas, USA [53]. This trial which is a first of its kind aims at testing whether DOTAP:Chol. nanocarrier-containing a TSG, Fus1, can be administered intravenously in patients with recurrent/metastatic lung cancer previously treated with platinum-based chemotherapy. Fus1 is a TSG located on chromosome 3p21.3 [54, 55]. The rationale for selecting Fus1 for NSCLC therapy is because it is frequently lost or deleted in more than 60% of patients diagnosed with lung cancer [56]. Additionally, studies have shown that Fus1 effectively suppressed lung-tumor growth *in vivo* when used as monotherapy or in combination with other drugs [57–59]. The primary objective of this trial is to treat patients with an escalating dose (0.01–0.09 mg/Kg) of Fus1-containing nanocarrier at a three-week interval and determine the maximum tolerated dose (MTD). Up to date, 23 patients have been entered on the study trial and have received one or more Fus1-containing nanocarrier treatment. Preliminary results demonstrate Fus1 nanocarrier treatment is well tolerated with no major treatment-related toxicity [53]. Furthermore, MTD is yet to be determined, and the trial is open and continuing to accrue patients. The final results of the Fus1 nanocarrier treatment is expected to be available upon completion of the trial. The outcome of this trial will facilitate the design of future TSG-nanocarrier-based Phase I/II clinical trials for lung cancer.

On the basis of our preclinical studies and the Fus1-containing nanocarrier Phase I clinical trial, two additional Phase I clinical trials for the treatment of pancreatic cancer, ovarian, and breast cancer have been approved by the Food and Drug Administration (FDA) (see Table 2). These trials will be conducted at the MD Anderson Cancer Center, Houston, TX, USA. Both of these trials have objectives and endpoints similar to the Fus1 trial. The only difference is the therapeutic gene to be used for these cancer types is different and not Fus1. For pancreatic cancer, a proapoptotic gene called Bcl-2 interacting killer (Bik) gene (BikDD) will be used for therapy [60]. The uniqueness is that BikDD gene expression will be under the control of cholecystokinin type A receptor (CCKAR) that will be conditionally regulated by VP16-GAL4-WPRE integrated systemic amplifier (VISA). This system is tumor selective and high BikDD protein expression is expected to occur in cancer cells with minimal protein expression occurring in surrounding normal tissues, and thus eliminating unwanted cytotoxicity. The objective of the pancreatic cancer Phase I clinical trial is to determine the MTD and optimal biological active dose (OBAD) compared with the clinical response. The trial has not been completed, and the results from this trial are therefore pending.

In the Phase I clinical trial planned for breast cancer treatment, the therapeutic gene to be incorporated into the DOTAP:Chol. nanocarrier is the E1A tumor suppressor gene. E1A gene introduction into breast cancer cells induces cell cycle arrest and cell death both *in vitro* and *in vivo* [61]. Additionally, E1A has previously been tested in a Phase I clinical trial for treatment of breast and ovarian cancer patients. Although results from the early trial did not show any therapeutic benefits, it demonstrated E1A treatment was safe [62]. This trial, like the pancreatic trial, is currently open for patient accrual and not completed. Therefore, results from this trial will remain unknown for, at least, the next one to two years. 

More recently plans for a Phase I clinical trial testing systemic IL-24 nanocarrier therapy for metastatic melanoma is underway. Preclinical efficacy and toxicity studies, that are prerequisites for submitting IL-24 nanocarrier as investigational new drug (IND), have been completed. The IL-24 nanocarrier phase I clinical trial is yet to receive approval from the Food and Drug Administration (FDA) and will be conducted at the MD Anderson Cancer Center, Houston, TX, USA.

It is evident from the number of Phase I clinical trials that have been initiated on the basis of our laboratory findings that the lipid-based DOTAP:Chol nanocarrier is useful for systemic delivery of cancer gene therapeutics. Successful translation of laboratory research to a clinic such as ours described above will lead to promising cancer treatment strategies and therapies. It is anticipated that additional laboratory research will be translated to the clinic in the next few years.

## 3. Conclusions

Since the inception and testing of gene-based therapies for cancer in the early 1990s, significant progress in the understanding of the biology of the disease and vector development has been made. Failure to control and/or eradicate cancer using virus-based cancer gene therapy has led to advancement of the nonviral delivery field. Despite skepticism and unexpected gene therapy related deaths, progress has been made in the area of cancer gene therapy and will continue to be made. It is evident from the progress made in our own laboratory, by moving laboratory research to the clinic one could successfully translate future research for cancer therapy. Since combination therapies have often been reported to produce additive to synergistic therapeutic effect, it is not far from testing Fus1 nanocarrier in combination with conventional chemotherapies or molecularly targeted therapies. For example one could combine Fus1 nanocarrier with the epidermal growth factor receptor (EGFR) kinase-targeted inhibitors for treatment of lung cancer. Similarly, IL-24 nanocarrier therapy can be combined with Raf-targeted inhibitor (sorafenib) or alternatively with the chemotherapeutic Temozolomide for treatment of metastatic melanoma. Given the possibility of testing various combinations, it is critical that the ongoing Phase I clinical trials are successful so that future clinical trials incorporating combination therapies can be designed and tested. 

With the advent of nanotechnology and its application to cancer medicine, novel nonviral vector-based nanocarriers that are multifunctional in their properties have been developed and are currently being tested in several laboratories around the world [24, 63–65]. It is thus not far from the near future that several Phase I clinical trials based on novel nanoformulations and properties will be initiated for testing drugs, genes, siRNA, aptamers, or molecular imaging agents for cancer diagnosis and therapy [27, 66, 67].

## Figures and Tables

**Table 1:** Tumor suppressor genes tested as cancer therapeutic in preclinical studies.

TSG	Cancer	Animal model	Therapeutic outcomes	Molecular events
E1A	Ovarian	Intraperitoneal tumor	Reduced abdominal tumor burden; increased animal survival	Apoptosis, reduced ascites, and cell cycle arrest
p53	Lung	Subcutaneous tumor; experimental lung metastasis	Tumor-growth inhibition; reduced extrapulmonary tumor nodules and increased animal survival	Cell cycle arrest, apoptosis, andantiangiogenesis
Fhit	Lung	Subcutaneous tumor; experimental lung metastasis	Tumor-growth inhibition; reduced extrapulmonary tumor nodules and increased animal survival	Cell cycle arrest and apoptosis
IL-24	Lung	Subcutaneous tumor; experimental lung metastasis	Tumor-growth inhibition; reduced extrapulmonary tumor nodules; increased animal survival	Cell cycle arrest, apoptosis, antiangiogenesis, and autophagy proimmune activity
Fus1	Lung	Subcutaneous tumor; experimental lung metastasis	Tumor-growth inhibition; reduced extrapulmonary tumor nodules; increased animal survival	Cell cycle arrest and apoptosis
BiKDD	Pancreas	Subcutaneous tumor; orthotopic tumor	Tumor-growth inhibition; reduced metastasis, increased animal survival	Apoptosis

**Table 2:** Synthetic nanocarriers tested for cancer gene therapy in human Phase I clinical trials.

Nanocarrier	Therapeutic gene	Cancer	Route of administration
DC (3 beta-[n-(N′, N′-dimethylaminoethane)-carbamoyl]cholesterol): DOPE (dioleoylphosphatidylethanolamine)	E1A	Breast/ovarian	Intratumoral (it)/intraperitoneal (ip)
DC (3 beta-[n-(N′, N′-dimethylaminoethane)-carbamoyl]cholesterol): Chol (cholesterol)	EGFR	Head & neck	Intratumoral
DOTAP (N-[1-(2, 3-dioleoyloxy)propyl]-N,N,N-trimethylammonium Chloride): DOPE (dioleoylphosphatidylethanolamine)	p53	Solid tumor	Intravenous (iv)
DOTAP (N-[1-(2, 3-dioleoyloxy)propyl]-N,N,N-trimethylammonium Chloride): Chol (cholesterol)	BiKDD	Pancreatic cancer	Intravenous (iv)
DOTAP (N-[1-(2, 3-dioleoyloxy)propyl]-N,N,N-trimethylammonium Chloride): Chol (cholesterol)	E1A	Breast/ovarian	Intravenous (iv)
DOTAP (N-[1-(2, 3-dioleoyloxy)propyl]-N,N,N-trimethylammonium Chloride): Chol (cholesterol)	Fus1	Lung	Intravenous (iv)
DOTMA (N-[1-(2,3-dioleyloxy)propyl]-N,N,N-trimethylammonium Chloride): Chol (cholesterol)	IL-2	Head & neck	Intratumoral (it)

Source: www.cancertrials.gov.
